# Economic analysis of new workplace technology including productivity and injury: The case of needle-less injection in swine

**DOI:** 10.1371/journal.pone.0233599

**Published:** 2020-06-17

**Authors:** Biaka Imeah, Erika Penz, Masud Rana, Catherine Trask, For the Needle-less Injector Study Team

**Affiliations:** 1 Canadian Centre for Health and Safety in Agriculture, College of Medicine, University of Saskatchewan, Saskatoon, Saskatchewan, Canada; 2 College of Medicine, University of Saskatchewan, Saskatoon, Saskatchewan, Canada; 3 Collaborative Program in Biostatistics, University of Saskatchewan, Saskatoon, Saskatchewan, Canada; Xiamen University, CHINA

## Abstract

Increasing intensification in swine production has led to new and specialized technologies, but the occupational health and safety impacts are rarely quantified in the business plans for adoption. Needle-less injection has potential to increase productivity and eliminate needle stick injury in workers, but it is not clear whether these benefits offset high capital investment and potential increases in musculoskeletal loads. This economic evaluation employed probabilistic scenario analysis using injury, cost, and production data gathered from interviews with swine producers in Manitoba and Saskatchewan. After adoption of needle-less injection, rates of needle-stick injury went down with no measureable effect on upper limb musculoskeletal disorders, resulting in lower health and safety costs for needle-less injectors. Needle-less injection duration was 40% faster once workers acclimatized, but large start-up costs mean economic benefits are realized only after the first year. The incremental benefit cost ratio promoted adoption of needle-less injectors over conventional needles for the base case of a 1200 sow barn; the conventional method is beneficial for barns with 600 sows or less. Findings indicate that well-designed technologies have the potential to achieve the dual ergonomics goals of enhancing human wellbeing and system performance. We anticipate that the economic and decision models developed in this study can be applied to other new technologies in agriculture and animal production.

## Introduction

Intensive agriculture is a farming system characterized by high productivity, high inputs of capital equipment, and high levels of output. As with other sectors of animal agricultural, swine production has become highly intensified. This intensification trend has led to fewer pig farms without a decrease in production. As an example, between 2001 and 2011, the number of pig farms in Canada decreased from 15,472 to 7,371(~53% decrease), but the total number of pigs produced stayed roughly the same (13.9 million vs. 12.6 million) [1]. The intensification of animal production promotes the specialization of workers into specific job roles and tasks, and the increased number of animals produced creates economic conditions that support acquisition of new technologies. As business-owners, swine producers who are faced with the option to implement new technology are likely to focus decision-making on reducing costs and increasing production. Occupational health and safety (OHS) can also have an impact on the fiscal health of an organization; the Shareholder Association for Research and Education states in a report that companies “may face great risks from failing to manage OHS issues” [2]. There is evidence that industrial intensification and its process changes may either increase existing musculoskeletal disorder (MSD) risk factors or introduce new ones [3–6]. Despite these findings, the ‘safety culture’ in agriculture remains low [7], and occupational health and safety may not be included in decision making. As livestock intensification may carry increased health and safety risks, there is a need to evaluate technological advances from the decision-makers’ (i.e. swine producers’) perspective in a way that includes health and safety. Evaluating technology decisions from a producers’ point of view would allow for development of policy or programs that can effectively enhance the health and safety of workers in this industry.

A relevant example of new technology adoption in intensive swine production is needle-less injectors. Modern swine production requires injecting pigs with vaccines, nutritional supplements such as iron [8], and antibiotics in case of illness. Before the introduction of the needle-less injector in the swine industry, conventional needles were routinely used to deliver all injections, with some drawbacks. The use of conventional needle injection comes with the risk of broken needles in animals, residual needle fragments in pork carcasses, transmission of infectious diseases between animals, and pork carcass defects [9, 10]. Broken needles can lead to metal fragments in meat and meat products, and are strictly guarded against in the pork value chain [11], often by euthanizing the animal. Pig carcass defects resulting from the use of hypodermic needle injection range from 2.7% hip bruises to 11.2% neck lesions [10]. In order to maintain consumers’ confidence in the industry, pork producers have developed numerous awareness campaigns within the industry on proper injection techniques for pigs. For example, a campaign in the early 2000s reinforced the idea among pork producers that one broken needle in any pig is too many [12]. In addition to pork carcass defects and other risks associated with the use of conventional needles, needle-stick injuries among pork production workers are inherent in needle use in the swine industry [13]. Needle-stick injuries occur when production workers accidentally puncture their own skin with needles, and represent an occupational hazard of conventional needles. In a survey evaluating the health and safety hazards associated with pig confinement facilities, 73% of survey respondents reported a needle-stick injury during their career [13].

As a newer technology replacing conventional needles, needle-less injectors are drug delivery technologies that do not use syringes and hypodermic needles. With needle-less injection, vaccines are delivered through a nozzle orifice via a high-pressure stream that penetrates the skin [14]. Needle-less injectors avoid some of the pitfalls of needle-syringe use: needle-stick injury, occupational exposure to blood pathogens, animal tissue damage, and animal stress [14]. Although needle-less injectors do help eliminate needle-stick injuries, concern has been raised among swine industry stakeholders of a potential to introduce new hazards, such as repetitive, forceful gripping or other postural or repetitive strain due to intensified and specialized task performance [15]. Studies have demonstrated effective immune responses to vaccine and supplements delivered by needle-less injection [16] and the effectiveness of needle-less injection in reducing pork carcass defects [17]. However, to our knowledge, no existing empirical study examines occupational health and safety (OHS) as part of producers’ decision making for needle-less technology adoption in swine production.

The objectives of this study are to: 1) describe the costs related to swine injection for both conventional needles and needle-less technology, including occupational health and safety impacts; 2) perform an economic analysis of needle-less injection versus conventional needle injection using data collected within multiple pig barns in the Canadian provinces of Saskatchewan and Manitoba.

## Materials and methods

### Context and data sources

This economic evaluation was conducted as part of a larger study examining the impact of technology adoption across multiple domains in the swine industry, with the goal of helping producers make decisions about technology adoption. The full study protocol is described elsewhere [15]. The comprehensive evaluation of needle-less injectors was intended as a case study for new technology adoption in animal production that considered occupational health and safety; it was developed with the participation of swine industry stakeholders. Needle-less injectors came into wide-spread use in the Canadian prairie provinces of Manitoba and Saskatchewan during 2010. Because there was a subsidy program administered by the pork producers associations during the year 2010, uptake was fairly rapid and those who were going to dopt this technology did so during the subsidy time period. It is estimated that the majority of the Canadian prairie swine herd is now raised in barns with needle-less injectors.

Data for the present study came from three sources: video data collected during the administration of injections to pigs to establish injection duration for both methods, Workers’ Compensation Board (WCB) claims statistics on needle-stick injuries and upper-limb musculoskeletal disorders before and after implementation of needle-less injectors, and a survey of cost data among swine producers.

### Video recordings of injection tasks

In order to quantify injection productivity with each method, video data were recorded from convenience samples of workers from two barns. Worker turnover can be quite high in this industry, so tasks with long training times to achieve full productivity have an impact on enterprise profitability. An expert user of a needle-less injector was defined as a barn worker that has been using needle-less injectors for more than a month. From barn 1 three full-time novice users of the needle-less injector participated, representing all workers who performed these injection tasks in the barn, From barn 2, 2 full-time expert users of the needle-less injector participated, representing 50% of the injection workforce in that barn. Unfortunately, it was not possible to identify any novice users of conventional needles within the current swine production workforce.

Informed consent from all participants was obtained prior to video collection. Ethics approval for this portion of the study was provided by the University of Saskatchewan Research Ethics Board (Certificate # 16–161).

Injection duration was determined using video analysis software (Noldus Pocket Observer 3.2, Noldus Information Technology). ‘Injection duration’ was defined as the time it takes to pick up a nursery pig or piglet, inject the pig with a vaccine, and put the pig back in the litter or pen. Injection duration was recorded for nursery pig vaccination and piglet processing; the duration for all the tasks involved in piglet processing were recorded, including docking tails, castration, and administration of supplemental iron. Vaccine administration via injection was the only task associated with the nursery pigs.

### Workers’ compensation board claim statistics

Data from the Workers Compensation Board from the Canadian Province of Saskatchewan include needle and syringe injury rates, upper limb musculoskeletal disorders (Carpal tunnel syndrome, rotator cuff syndrome, Tendonitis, and Tenosynovitis) injury rates specific to the swine industry (sector A1114 ‘piggery’); this data includes all swine production enterprises with an employer-employee structure, but not small family farms which have the option not to enroll in WCB programs. The dataset included historical trends in injury rates from 2004 to 2016. Implementation of needle-less injectors occurred in the province of Saskatchewan in 2010. The average annual injury rates were calculated for the years 2004–2009 (‘pre-implementation’) and for the years 2011–2016 (‘post-implementation’).

### Cost survey

A questionnaire designed to collect data on the cost components of needle-less injector and conventional needle injector use was administered to 10 key informants from 5 swine industry organizations in Manitoba and Saskatchewan, including large corporate farms with multiple barn locations, colony farms, and large privately-held farms. Collectively, it is estimated that this sample covered over 50% of the Swine herd in the prairie provinces of Manitoba and Saskatchewan. The cost categories obtained through the survey are described in Table 1. In cases where the primary contact didn’t have all the needed cost information, a snowball sampling technique was used to identify additional informants. The process continued until the requested data had been collected for each enterprise. This portion of the study was exempted from Ethics review by the University of Saskatchewan Research Ethics Board (Certificate # BEH 17–10).

**Table 1:** Calculations, data sources, and assumptions associated with fixed and variable costs for needle-less and conventional needle injector evaluations.

Cost Description	Needle-less injector	Conventional needle
**Start-up cost of equipment (fixed)**	Mean purchase price ($) of items in year 1.	None
Initial purchase price of needle-less injector unit, maintenance tools, and compressor unit (if required), as reported by Saskatchewan & Manitoba swine producers		
**Training cost (variable)**	Novice workers/yr * wage($) * (trainer hrs * + additional injection time in hrs * 1 month of working hrs)	None
Based on turnover rate (i.e. number of novice workers/yr) and trainer cost (i.e. wage and hours) as reported by swine producers. Producers reported ‘on the job training’ where novices perform productive work at a slower pace. Reduced productivity via video comparison of novices and experts.		
**Maintenance (variable)**	Replacement parts/yr ($) + wage($) * (52*Weekly maintenance hrs + 312 *daily maintenance hrs)	Injection tools are disposable, no maintenance
Cost of consumable supplies and parts needed to maintain the needle-less system (e.g. replacement seals, cleaning fluids, etc.) and the daily and weekly labor hours as reported by Saskatchewan & Manitoba swine producers		
**Needles and Syringes (variable)**		needles+syringes ($) *injections/pig* pigs produced/ yr
Consumable/disposable needles and resuable animal syringe tools as reported by Saskatchewan & Manitoba swine producers		
**Disposal (variable)**	No special disposal required	Annual cost ($)
Annual cost associated with hygienic disposal of needles and syringes as reported by Saskatchewan & Manitoba swine producers		
**Injection task (variable)**	Wage ($) * injection duration *pigs injected per yr	Wage ($) * injection duration *pigs injected per yr
Productivity (task completion time) of each injection method determined via video comparison of novices and experts. Wage rate as reported by Saskatchewan & Manitoba swine producers		

### Economic analysis

#### Incremental benefit-cost analysis

In CBA, all benefits and costs need to be identified and expressed in monetary terms. This allows for direct comparison of costs and benefits and for the results of the analysis to be expressed in terms of a cost-benefit ratio, benefit-cost ratio, or net benefit (net present value) resulting from the intervention. For a CBA expressed as net benefits, the decision rule states that an intervention with a positive net benefit is worthwhile and should be undertaken (if evaluating a single intervention) and when comparing multiple interventions, the one with the highest net benefit should be undertaken. To use benefit-cost ratio to choose between competing interventions (mutually exclusive), we must evaluate the incremental cost and benefits associated with adopting the new technology. For a CBA expressed as incremental benefit-cost ratio (IBCR), the focus is on the marginal cost and benefit associated with the introduction of a new intervention. IBCR is mathematically expressed by dividing the discounted incremental benefit of the new technology, by the discounted incremental cost:

$$IBCR=\frac{B_{1}\;-\;B_{0}}{C_{1}\;-\;C_{0}}\;\geq 1$$

Where:

IBCR = Incremental benefit-cost ratio
B_1_ = Discounted benefit of new intervention
B_0_ = Discounted benefit of alternative
C_1_ = Discounted cost of new intervention
C_0_ = Discounted cost of alternative

The IBCR is an extension of the benefit-cost ratio. The IBCR accept/ reject criteria is to accept new interventions that cost more than the base case if IBCR>1 [18]. IBCR >1 implies every additional dollar spent on the technology increases net benefit by more than one. If the IBCR is less than one, then the technology with the lower cost should be adopted. This criterion only applies with the numerator and denominator of IBCR being strictly positive [18]. When the denominator (numerator) is negative, the less (more) costly alternative should be adopted [18]. Since the decision to adopt new technology lies with the swine producers, the economic analysis was performed from their perspective. Benefits were measured as the number of pigs saved each year from being euthanized as a result of broken needle. Costs included equipment start-up cost, equipment maintenance cost, training cost, labour, and needle disposal cost. Cost and benefits are reported in 2017 Canadian dollars. Future benefits and costs are discounted according to the CADTH guideline for economic evaluation research [19]. Two time horizons were adopted for the benefit-cost model: the short-term horizon was nine years (selected since this was the manufacturer-estimated lifetime of the needle-less injector); and the long-term time horizon was 27 years (selected to represent long-term us of the technology, and which incorporates replacement of the needle-less injector 3 times).

#### Swine producer’s cost

Direct labor cost is determined by multiplying the time spent injecting a pig, with either of the injectors, by the wage rate of barn technicians. The wage rate of barn technicians does not change on introduction of this technology. Future costs are converted to their present values at the 4% interest rate in the base case scenario. Costs are reported in 2017 Canadian dollars. Table 1 details the costs associated with the needle-less and conventional needle injectors.

Since all the barns in our study had adopted needle-less injector and conventional needle is only rarely used for specific treatments (for example, antibiotics to treat infection), there was no available data on the number of needles used in a barn that uses solely conventional needles to inject pigs. The number of needles was therefore estimated using information gathered in the questionnaire, and calculated as follows:

$$\frac{\left(Number\;of\;pigs\;produced/year\right)*\left(Number\;of\;injections/pig/lifetime\right)}{Number\;of\;injections/needle}$$

#### Benefit

Broken needles found in pig carcasses are rare, but a single occurrence is a serious food safety hazard. In an effort to prevent broken needles fragments from getting to consumers, many pork producers have started converting their swine barns to needle-less injection. Our questionnaire for collecting cost data included a section on broken needles, including estimates of the frequency of broken needles and the protocol for handling broken needles. Generally, a broken needle that cannot be recovered results in a total loss of the animal (which is euthanized) and reduction in the productivity of the barn. To evaluate incremental benefit of needle-less injectors for the base case, we used the median number of broken needle occurrence in a year (i.e., the number of pigs that would not be lost when switching to needle-less technology). Benefit was determined by multiplying the number of broken needles each year by the price of a market pig. We reported cost and benefit in the base year, 2017.

#### Sensitivity analysis

Injection duration is dependent on the expertise of users, i.e. expert vs. novice. The proportion of novice versus expert users was used to calculate the weighted mean duration of injection administered using needle-less injector or conventional needle syringe. In the base case, the proportion of novice and expert users was 25% and 75% (as reported by swine industry stakeholders). This proportion is variable, since higher labor turnover rate will increase the proportion of novices. Extreme case comparison was also performed (100% novice and 0% expert as well as 0% novice and 100% expert) to evaluate whether the type of user significantly affected results of the analysis. We also conducted a sensitivity analysis of the impact that the choice of discount rate will have on the results.

In order to evaluate the robustness of our findings in relation to potential bias, unmeasured confounding, and uncertainty in the observed data, we quantitatively evaluated by probabilistic bias analysis [20]. Instead of specific values for different cost and benefit components of each injection method, we chose uniform distribution with observed minimum and maximum values as the range of distribution. It should be noted that most inputs to the cost and benefit calculation are limited to a set of range values (minimum and maximum) based on small convenience and snowball samples. The uniform distribution reflects a wide range of variability in the incremental benefit-cost ratio calculation without imposing any strong assumption on the poorly known input variables [21, 22]. In some cases, the observed quantity (i.e. needle-stick injury claim cost or sharp object disposal cost) was constant; we varied such quantities by 20% around the observed values, an arbitrary number consistent with other examples in the literature [23]. The uniform distribution reflects uncertainty about the input values on total cost and total benefit for each injection method with equal probability of selection for all values within the range. The uniform distribution, along with input variation, makes our analysis stochastic to incorporate inherent randomness in the behavior of incremental benefit-cost ratio [21]. We reported multiple bias scenarios: all input variables were allowed to vary probabilistically within their intervals, as well as single bias scenarios: the sample mean as an input for a particular variable. Single bias scenarios included expert users, novice users, small farm (barn size 300 pigs), and large farm (barn size 3000 pigs). These barn sizes were selected to represent the size range of commercial barns on the Canadian prairies. We summarized cost and benefit distributions for each injection method using mean and 95% simulation interval based on 5000 iterations of probabilistic bias analysis. This analysis was performed using R. The mean and intervals, thus obtained, are considered equivalent to conventional point estimate and frequentist confidence interval, respectively [20]. The results are graphed as an incremental cost benefit plane and cost effectiveness acceptability curve (CEAC) [24].

## Results

### Video analysis

Video recordings were completed for 434 injections (297 from barn 1 and 137 from barn 2) of nursery pigs and 302 injections (from barn 1 only) of piglets. Total hours of recorded video were 1.4 and 3 hours for nursery pigs and piglets, respectively. For piglets, injection duration differs between female and male piglets due to castration of male piglets. Table 2 shows the mean injection duration in seconds for nursery pigs and piglets.

**Table 2:** Injection duration per pig, in seconds, for two swine injection tasks by novices & experts.

*Injection Methods*	Worker Expertise	Pig Type	Number injected pigs	Mean Injection duration	Range	Standard deviation
Needle-less	Novice	Nursery pigs	149	9.8	2.4–30	3.5
Needle-less	Novice	Piglets	161	30.2	12.1–86.2	12.6
Needle-less	Expert users	Nursery Pigs	137	4.2	1.4–15.3	2.1
Needle	Expert users	Nursery Pigs	148	9.6	3.3–25.3	2.7
Needle	Expert users	Piglets	141	28.0	12.9–63	11.4

*note that it was not possible to recruit swine barn workers who were novice users of conventional needles or expert users who performed piglet injection.

### Summary of cost estimates

Table 3 shows the cost summary for needle-less and conventional needle injectors. Fixed costs applied only to the needle-less injectors; in addition to the injector unit itself there were costs for maintenance tools and a compressor. There are two kinds of needle-less injectors: compressed gas powered and battery powered. The compressed gas-powered needle-less injector version was used by the surveyed producers and requires an electric compressor to operate, indicated as a fixed cost.

**Table 3:** Cost components of needle-less injector and conventional needles, as estimated for a 1200-sow barn.

Cost Item	Needle-less Injector (CAD $)		Conventional Needle Injector (CAD $)	
	Mean	Range	Mean	Range
**Fixed costs**				
Needle-less Injector Unit	4834	3107–5854		
Maintenance tools	431	0–1000		
Compressor	409	0–798		
**Variable costs**				
Training (per new worker)	120	100–140		
O-rings replacement cost (per yr)	198	2.60–785		
Daily maintenance labour (per yr)	2135	548–6570		
Weekly maintenance labour (per yr)	507			
Cleaning Supplies (per yr)	59	0–122		
Other repairs (per yr)	768	460–1367		
Disposable needles (per yr)			3974	3596–4637
Disposable syringes (per yr)			5400	150–20625
Sharp object disposal (per yr)			26.5	
Injection task labour (per yr)	7091		8252	

Variable costs depend on the number of pigs and the timeframe under evaluation; Table 3 also shows variable costs based on a 1200-sow barn, which is the median size of surveyed operations. Routine maintenance on the needle-less injector is vital to ensure safe operation and longevity. Some of the barns reported purchasing separate maintenance tools for needle-less injector maintenance. Daily routine maintenance, such as lubrication of the amplifier (CO_2_ regulator), and flushing the fluid path, were performed on a daily and weekly basis and took 19.5 minutes on average. Weekly maintenance included inspection and replacement of any fatiguing parts, including O-ring seals, and took an average of 32.5 minutes. This translated into an annual (average) labor cost of $2642 ($2,135 for daily maintenance and $507 for weekly maintenance). Labour saving reflected by the decrease in injection duration for needle-less injector resulted in a cost reduction of $1,161 per year.

### Cost of injuries

Since direct injury claims costs are borne by WCB insurance, the main injury cost from the employer’s perspective results from productive time loss when replacement workers must be recruited and trained to replace workers on disability leave. The average annual needle-stick injury rate before needle-less injector implementation was 2.13% claims per full-time equivalent workers covered per year, after implementation it was 0.74%. WCB claims statistics indicate that needle-stick injury claims include health care costs only, with no loss in productive work time in the form of absenteeism [25]. Since healthcare costs are borne entirely by the Workers’ Compensation system, there were no employer-borne costs associated with needle-stick injury. The average annual upper-limb musculoskeletal injury rate before needle-less injector implementation was 0.15% claims per full-time equivalent workers covered per year, after implementation it was 0.07% [25]. As with needle-stick injuries, the healthcare costs are paid by the Workers’ Compensation system. The average lost work time for upper limb musculoskeletal claims is 26 days, and Workers’ Compensation also pays for the wage replacement for the recovering worker. However, during this time a replacement worker would need to be recruited and trained from novice to expert status. Therefore, the main employer-borne costs of time loss is the hiring and training an additional worker whose reduced ‘novice’ productivity impacts overall labour costs, therefore injury costs are reflected in Table 3 as ‘training’, which are described further in the sensitivity analysis.

### Cost-benefit analysis

The benefit of needle-less injection was conceived here as the number of pigs saved from being euthanized as a result of broken needles (reported by producers as an average of 1.23 pigs per year per 1200 sow barn). Table 4 shows the discounted costs based on empirical findings; this translated into an incremental benefit of $1840 for the needle-less injector relative to using conventional needles. The incremental cost is negative (- $13,059), meaning that the needle-less injector is less costly over a 9-year life of the system, largely a result of labour savings. The incremental cost and benefit result in a negative IBCR of -0.14. There are two cases when IBCR is negative: (1) the new technology is more costly and offers less benefit (numerator of the ratio is negative); (2) the new technology is less costly and offers more benefit (denominator of the ratio is negative). The second case applies to the needle-less injector and according to the IBCR acceptance criterion, the needle-less injector should be adopted when framing the two injection types as mutually exclusive.

**Table 4:** Base case incremental cost, incremental benefit and incremental benefit-cost ratio (IBCR) over the 9-year life span of the needle-less injector (in discounted 2017 dollars).

Incremental Benefit of needle-less injector	$1840
Incremental Cost of needle-less injector	$-13,069
IBCR of needle-less injector	$-0.14

### Probabalistic bias analysis

Results from the probabilistic bias analysis have been summarized in Table 5. Results from the probabilistic bias analysis indicated that over both a 9-year and 27-year time frame, the needle-less injector method became less costly and more beneficial than the conventional needle method.

**Table 5:** Cost, benefit, and net benefit of injection methods and their 95% simulation intervals from probabilistic bias analysis mean (95% CL) based on 5000 iterations.

	Needle-less Injector	Conventional Needle
**Base year**		
**Cost**	$14,377 (9,470–20,152)	$7,778 (477–15,232)
**Benefit (in100k)**	$58.97 (3.18–124.39)	$58.96 (3.18–124.39)
**Short-term (9-years)**		
**Discounted Cumulative Cost (2017 dollars)**	$53,380 (43,723–63,182)	$66,996 (39,246–95,229)
**Discounted Cumulative Benefit (in100k and 2017 dollars)**	$509.89 (297.43–733.27)	$509.87 (297.41–733.25)
**Incremental Benefit-to-cost ratio [first quartile, third quartile]**	-0.11 (-1.35 to 1.39) [-0.18, -0.06]	
**Long-term (27-years)**		
**Discounted Cumulative Cost (2017 dollars)**	$53,495 (44,066–63,295)	$66,622 (38,908–95,089)
**Discounted Cumulative Benefit (in 100k and 2017 dollars)**	$507.14 (291.27–736.56)	$507.12 (291.25–736.54)
**Incremental Benefit-to-cost ratio [first quartile, third quartile]**	-0.06 (-1.45 to 1.32) [-0.18, -0.05]	

**Fig 1:**
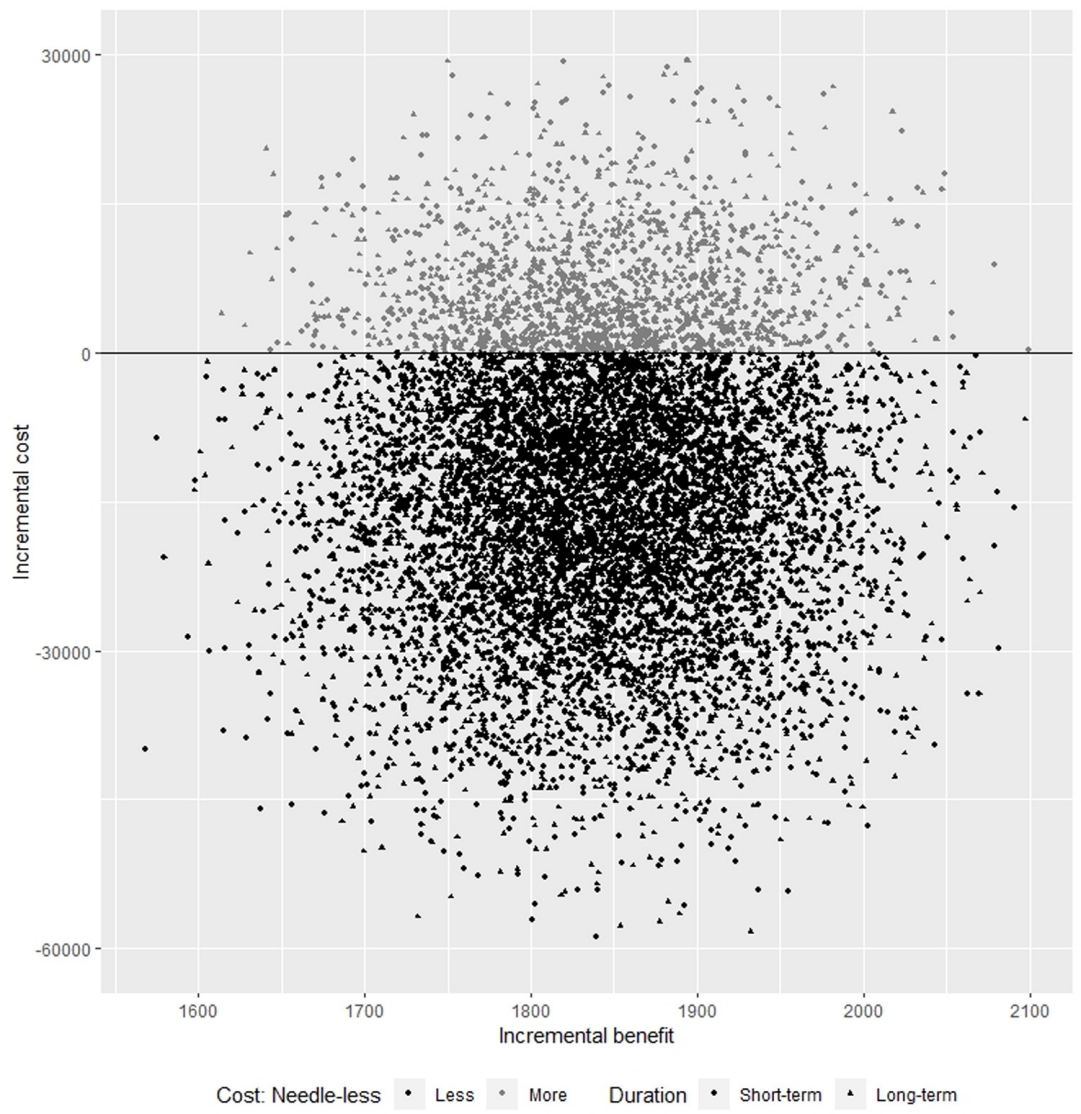
Sensitivity analysis: Cost effectiveness plane showing incremental cost vs incremental benefit over 9-years (circles) and 27-years (triangles) for a switch from conventional needles to the needle-less injector, based on probabilistic bias analysis with 5000 simulations.

Fig 1 shows the findings from single bias scenarios. While incremental benefit is always positive (indicated by positive values on the X axis), the incremental cost is most frequently negative (i.e. dark data points below the 0 cost line), indicating that the switch to needle-less injector costs less in most scenarios.

**Fig 2:**
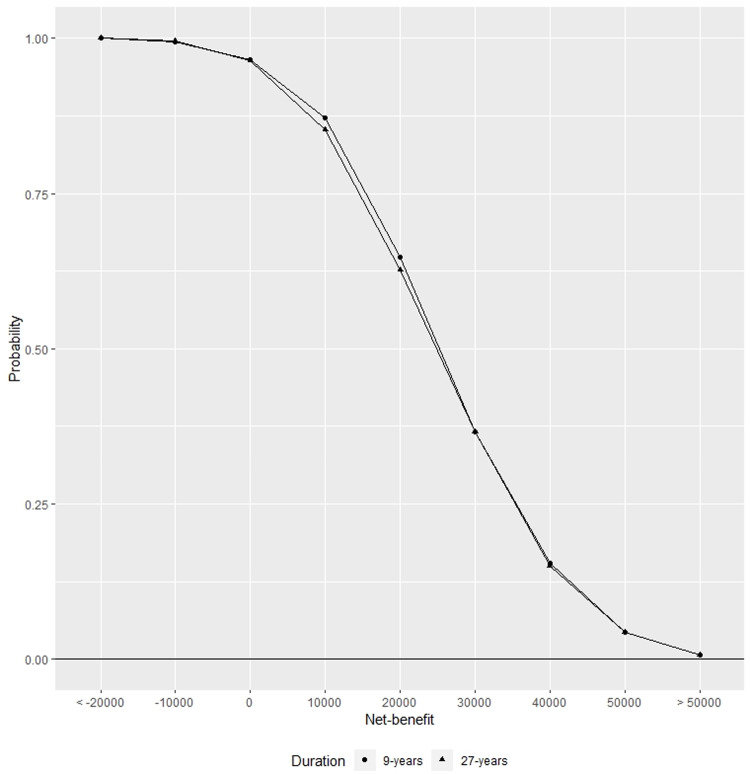
Cost effectiveness acceptability curve (CEAC) over 9-years (circles) and 27-years (triangles) for a switch from conventional needles to the needle-less injector.

Fig 2 shows the cost effectiveness acceptability curve (CEAC) derived from the sensitivity analysis. Although it is not strictly correct to interpret this as the probability of the needle-less injector being cost-effective [24], inspection of the costs findings indicate the needleless method is most frequently cost-saving, as it intersects the Y-axis at approximately 1. Inspection of the benefit findings show the needleless method is nearly always beneficial in terms of a positive net-benefit, since the CEAC asymptotes towards 1 at zero net-benefit.

### Sensitivity analysis

**Fig 3:**
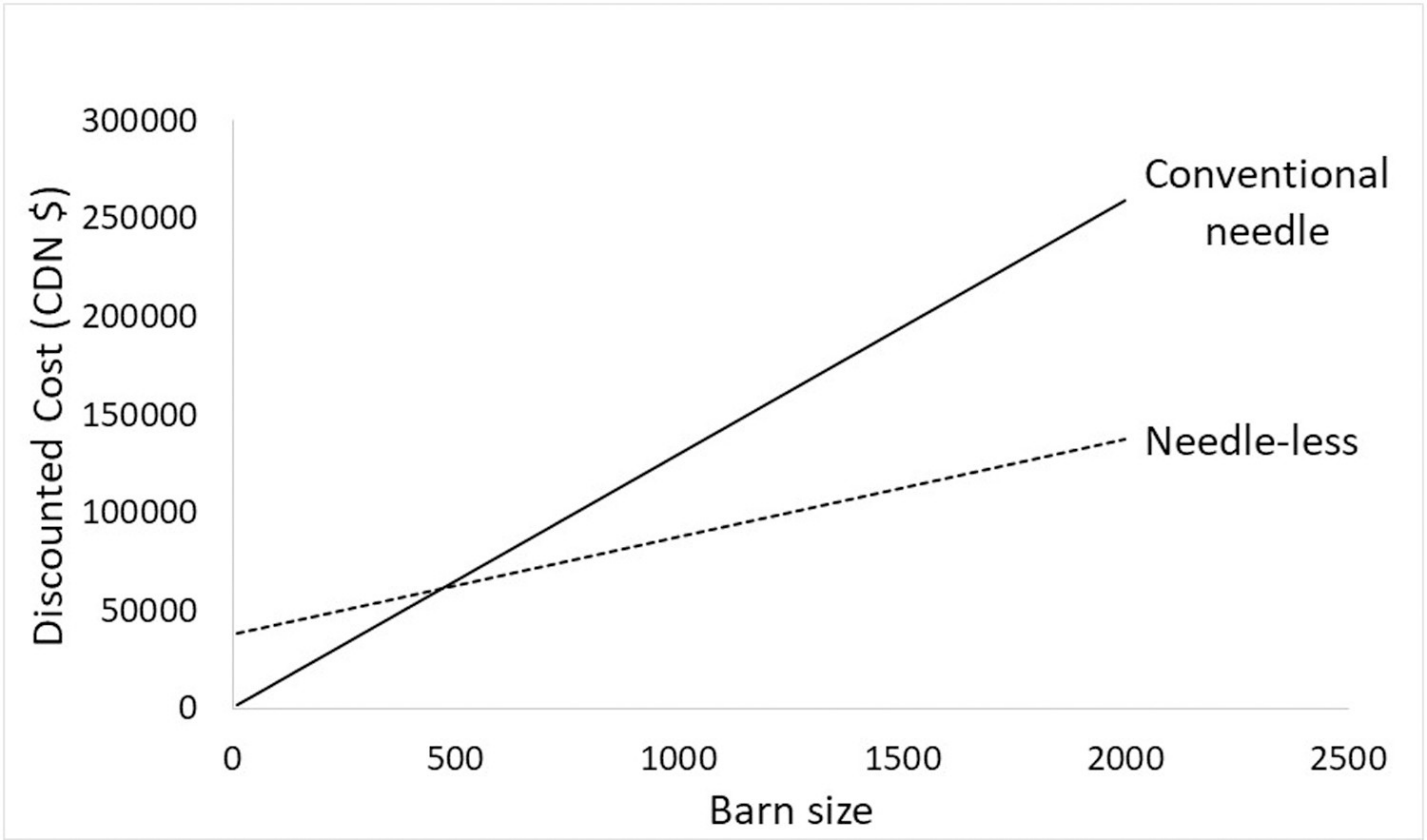
Net present value of total cost for needle-less (dashed line) and conventional needle (solid line) swine injection by barn size.

We performed one-way sensitivity analyses to determine how changes in key variables impact the costs and benefits of needle-less and conventional needle injector. Barn size and interest rates were varied across a plausible range of values with all other variables constant at their average values. The lower and upper bound for barn size were 10 farrow-to-finish pigs and 6000 farrow-to-finish pigs respectively. The total discounted costs range for one needle-less injector for a 10–6000 farrow-to-finish barn size is $26,029 to $111,339. Total discounted cost (benefit) of a 1200 farrow-to-finish barn that uses needle-less injector is $50,000($2,376.82) at 0% interest rate and $31,065(1,1341.62) at 15% interest rate. The total discounted cost for conventional needle is $126,793.9 at 0% interest rate and $71,869.3 at 15% interest rate. A 1200 farrow-to-finish barn may require more than one unit of the needle-less injector system. Therefore, the discounted cost above could double ($100,000 at 0% and 63,302 at 15% interest rate) for that barn size. While holding the interest rate constant (at 4%) and all other variables constant at their median values, we varied the barn size across a plausible range of values then calculated the discounted cost for needleless and conventional needle over the lifespan of the needless system. The results suggest that a farrow-to-finish barn size of less than 600 would be better off using conventional needle. Fig 3 shows the cost of both injector types over a range of barn sizes.

## Discussion

Conventional needles have long been used in the swine industry, but more recently needle-less technology has been increasingly adopted. However, to our knowledge, it has not been previously reported whether the benefits outweigh the costs associated with this newer technology, especially when considering worker health and safety outcomes. Our findings demonstrate that the incremental benefit-cost ratio promotes adoption of needle-less injectors over conventional needles in our base case (barn size of 1200-sow farrow to finish) over the short- and long-term time frames of 9 and 27 years, respectively; we further show that the ICBR supports needle-less injector adoption for barn sizes greater than 600 sows.

Most of the value of needle-less injectors arises from reduced labour cost. Our study found needle-less injection device was 2.3 times faster than a conventional needle for injecting a nursery pig. The time to inject one nursery pig with a needle-less injector averaged 4.21s and was faster than a conventional needle-syringe, which averaged 9.58s. The reduction in time with the needle-less injector is consistent with previous findings in other areas of animal production; Mousel et al. found that the time to inject 7 to 9 sheep averaged 60.6s for pneumatic, needle-free injector and 155.3s for needle [26]. Using a conventional (sharp) needle may require more re-positioning to ensure that the injection angle is favourable, and may require more caution to avoid breaking a needle or the worker incurring a needle stick. Another positive feature of the needle-less injection method was reduced loss of saleable pork due to broken needles. Previous studies have shown that needle-less injectors are likely to reduce injury at the injection site when compared to conventional needles [17]. In the present study, the rate of pork carcass condemnation as result of broken needles averaged 1.23 per year per 1200 sow barn in the barns that used conventional needles, compared to zero reported losses due to needle-less methods. This has implications in a sector that continues to demonstrate international growth. In the 10 years between 2008 and 2018, US pork production expanded from 117 to 125 million head [27]. During this timeframe pork production rose in China from 602 to 694 million head, in the EU from 303 to 327million head, and in Brazil from 32.70 to 42.64 million head [27]. Although data on the uptake of needle-less injectors was not available, it might be reasonable to infer that higher-income nations have more coverage than lower-income nations, and that the decision to adopt the technology is still a timely one for many producers.

The current study did not examine the serological responses of pigs to both injection methods; a fundamental assumption of our evaluation is that both methods are equally effective in terms of delivering medication and physiological response of the animal. This assumption is supported by several previous studies that have examined the efficacy of needle-less injector on antibody production, both in beef calves [16] and in pigs [17]. Needle-less methods have also been shown to be equivalent to conventional needles as a precursor for swine stress levels [28], important in fostering a thriving herd.

Occupational health and safety impacts such as workplace injuries can increase the cost of implementing a new technology like needle-less injectors. Sometimes a new technology can solve one problem and introduce another. In the case of swine injection, the main injury types we considered were needle-stick related injury and upper limb musculoskeletal disorders (e.g. carpal tunnel syndrome). Although there is a clear mechanism for needle-less injectors to reduce needle-stick injuries, it was hypothesized they might introduce new hazards, such as higher repetition, more forceful gripping, or postural strain due to intensified task speed. In dairy farming, industrial intensification has been shown to change musculoskeletal exposure profile [3, 4], as well as to increase [5] or change the location [6] of reported musculoskeletal disorders (e.g., from the knees to the back). Swedish dairy workers, for example, demonstrated increasing rates of MSD from 83% in 1988 to 90% in 2002 [5]. This change was concurrent with increased task time and musculoskeletal exposure duration using modern milking equipment [5]. However, the present study did not find any increased risk of upper limb musculoskeletal disorders after introducing needle-less injectors; in fact, we found there was a small reduction (0.15% to 0.07% per 100,000 full time equivalents or ‘FTE’ per year). Needle-stick injuries also were reduced, decreasing from 2.13% per to 0.74% per 100,000 FTE per year. In swine barns, the introduction of needle-less injectors does not eliminate conventional needles since there is still need for some needles in the barn for one-off treatments that are not appropriate for the needleless injector; therefore the needle-stick rate does not go to zero.

### Strengths and limitations

Our calculation of ICBR assumes that needle-less and conventional needle are mutually exclusive interventions for the work tasks of interest; i.e. implementing needless-less injectors means that conventional needle would not be implemented. While this is true for routine injection tasks, there is still occasionally need for small disposable conventional needles for non-routine/infrequent administration of specialized medicines for sick animals.

The costs associated with occupational injury and illness can be substantial, and it has long been reported that the indirect costs of musculoskeletal disorder can exceed the direct costs [29, 30], only a portion of which are borne by employers. As the present analysis considered the employer’s perspective, the societal-level direct and indirect costs are not included. In many jurisdictions, direct health care costs arising from workplace injuries and long-term leave to recover are generally paid for by WCB insurance and so not paid directly by the employer. However, if a worker takes long-term leave, an employer may need a new worker to replace them, and that worker would need to get trained. Alternatively, if a worker takes a disability leave, other workers may be asked to work overtime, and may be paid a higher wage rate (i.e. time and a half) for those hours. These indirect personnel costs are included in the present analysis since they are borne by the employer. Indirect costs become harder to quantify when they are related to productivity decrements related to working while ill or injured, a phenomenon that has been called ‘presenteeism’ [31]. Qualitative investigation of agriculture sector workers demonstrates a substantial burden of musculoskeletal pain symptom-related reduction in quality of life, reduced contributions to community and family activities, and reduced participation in leisure activities [32, 33]. These costs would be borne by the worker and although they can have a substantial impact, they are also not included in this analysis. There is undoubtedly social benefit derived from reducing workplace injury cost that is borne by insurance, but this cost is not currently ‘owned’ by swine producers and so does not factor in their economic decision-making.

Short of large-scale changes to national regulation and insurance policy changes, the most feasible opportunities for intervention seem to be those which influence employer decision making.

To our knowledge, this is the first published study to evaluate the economic impact of needle-less injectors using real data collected from multiple swine barns and including the employers’ perspective of health and safety considerations. This study used an employer perspective on costs and benefits and incorporated domains of high ergonomic importance, including productivity and the employer-owned costs of injury. Although the sample size for data collection was not large, the enterprises included in the study represent over 50% of the total herd in the sampled provinces and include a range of barn sizes and organization types.

The variability of the estimates was also expressed via 95% simulation intervals during sensitivity analyses. Systematic error (bias; e.g. confounding, misclassification, selection bias) is a potential source of flawed findings in observational studies; the susceptibility is even higher with claims data [34], or a small sample. Quantitative evaluation of systematic error is more impactful than random error in such studies [35, 36], and a considered a best practice for studies generating policy recommendations [37]. The present study reports 95% simulation intervals from probabilistic bias analysis around costs and benefits, providing a measure of variability around our estimates which may relate to random error, systematic error, if any, and uncertainty in the data parameter values that may influence the true net benefit. [20]. Moreover, we report multiple bias scenarios, where all variables of interest are permitted to vary probabilistically within their intervals, as well as single bias scenarios with a specific value for a particular variable (in this case, barn size and expert vs novice users).

However, there were also a number of limitations to this study. In cost modeling, we assumed that the barn size stays the same over the useful life of the needle-less injector (9 years). Such assumptions are likely to lead to conservative estimates of cost and benefit, especially since the cost of needles and syringes varies with the size of the barn. Including variation in barn size between measurement periods may affect overall net benefit of needle-less injector compared with the conventional needle injection. Additionally, the cost survey was carried out using a convenience sample within two provinces, so mean costs are unlikely to be fully representative. In general, convenience sampling can lead to either under-representation or over-representation of a particular group within the sample. Given the retrospective nature of the injury data and the fact that it aggregates all enterprises in the swine sector, there are no contemporaneous control groups (i.e. groups not exposed to needle-less injectors). Therefore, it is not possible to distinguish effects (or lack thereof) due to the needle-less injector and effects related to other policy, technology, or market changes which took place during the 10-year dataset timeframe. We did not directly adjust for any variation (i.e. regional or policy) in the cost component of both needle-less and conventional needle injector. However, we anticipate regional and temporal variation will have minimal effect on our conclusion since we employed distributions instead of singular values for each component of cost in our probabilistic bias analysis. Although it is always prudent to be cautious when generalizing results, our findings are potentially relevant to a very large population. About half of the world’s labour force is involved in agriculture [38], and 40% of global agriculture economic outputs are related to livestock production [39]. As worldwide trends of agricultural intensification appear to be accelerating rather than abating [39], the question of whether to adopt new technology (and the related impacts on worker health) becomes important to a greater portion of this workforce.

Another limitation is that we didn’t record video for expert users giving needle-less injections to sows or newborn piglets, and therefore cannot estimate productivity differences as we did for nursery pigs. If the savings are similar, then our productivity benefit estimates are likely conservative since the injection labour is duplicated during a pig’s lifetime. Further, since all the barns in our study adopted needle-less injection and conventional needle is used for specific treatment, we didn’t have access to data on the number of needles used (per year) in a barn that uses only conventional needle to inject pigs. We had to estimate this number given available information about barn size, number of injections per needle, and the number of pigs produced per year. However, we performed robust sensitivity analyses to account for our lack of confidence in our model estimates. Future studies could investigate operations that have adopted needle-less injector versus those that have yet to adopt it. Future studies could also take the approach of comparing pre-adoption and post-adoption numbers of worker needle-stick injuries and pork carcass condemnation related to conventional injection. In the present study we made the decision not to include WCB claims cost, since it is not borne by the producers directly. Most workers’ compensation systems (including in the provinces of Manitoba and Saskatchewan) calculate insurance premiums in two ways: 1) the industrial sector rating which is the same for all enterprises in a sector and reflects the average risk of that sector; and 2) the experience ratings, calculate on a multi-year moving average to reflect the ‘safety performance’ of an enterprise relative to their same-sector peers. We deemed this appropriate, since an increase in claims of 1.34 per year (in the case of needle-stick injury) or 0.08 per year (in the case of upper limb claims) seem unlikely to have a meaningful impact on the experience rating, which contributes only part of the total insurance premiums for an enterprise.

## Conclusion

This study presents the results of an economic evaluation of needle-less injector compared with conventional needle in delivery of vaccine, nutritional supplements and antibiotics to pigs from the swine producers’ perspective. When considering those costs and benefits owned by swine producers, we found the net benefit of needle-less injection to be slightly higher than that of conventional needle and likely to be the case for barns with more than 600 sows. Due to large start-up cost associated with needle-less injector, the benefits of this injection method is seen beyond 1 year of use. This study provides some initial footing for future research of the cost-benefit of introducing new technologies in intensive agriculture. Further, it provides some insight into those occupational health and safety impacts of new technology that may influence the decision making of swine producers. Further analyses that include several barn sizes and barns that are yet to adopt needle-less injector would fill gaps left by the limitations of this study.
